# Liver sinusoidal endothelial cell ICAM-1 mediated tumor/endothelial crosstalk drives the development of liver metastasis by initiating inflammatory and angiogenic responses

**DOI:** 10.1038/s41598-019-49473-7

**Published:** 2019-09-11

**Authors:** Aitor Benedicto, Alba Herrero, Irene Romayor, Joana Marquez, Bård Smedsrød, Elvira Olaso, Beatriz Arteta

**Affiliations:** 1 Department of Cellular Biology and Histology, University of the Basque Country, School of Medicine and Nursing, 48940 Leioa, Bizkaia Spain; 2 Department of Medical Biology, Vascular Biology Research Group, University of Tromsø, Tromsø, Norway

**Keywords:** Gastroenterology, Oncology, Pathogenesis

## Abstract

The prometastatic stroma generated through tumor cells/host cells interaction is critical for metastatic growth. To elucidate the role of ICAM-1 on the crosstalk between tumor and primary liver sinusoidal endothelial cells (LSECs) and hepatic stellate cells (HSCs), implicated in tumor adhesion and angiogenesis, we performed *in vitro* cocultures and an *in vivo* model of liver metastasis of colorectal cancer (CRC). ICAM-1 blockade in the LSECs decreased the adhesion and transmigration of tumor cells through an LSEC *in vitro* and *vivo*. Cocultures of C26 cells and LSECs contained higher amounts of IL-1β, IL-6, PGE-2, TNF-α and ICAM-1 than monocultures. C26 cells incubated with sICAM-1 secreted higher amounts of PGE-2, IL-6, VEGF, and MMPs, while enhanced the migration of LSECs and HSCs. HSCs cultures activated by media from C26 cells pretreated with sICAM-1 contained the largest amounts of VEGF and MMPs. C26 cell activation with sICAM-1 enhanced their metastasizing potential *in vivo*, while tumor LFA-1 blockade reduced tumor burden and LSECs and HSC-derived myofibroblasts recruitment. *In vivo* ICAM-1 silencing produced similar results. These findings uncover LSEC ICAM-1 as a mediator of the CRC metastatic cascade in the liver and identifies it as target for the inhibition of liver colonization and metastatic progression.

## Introduction

Colorectal cancer (CRC) represents one of the most prevalent malignancies worldwide^1^. Primary lesions are usually resectable, whereas prognosis worsens when distant metastases are detected. As many as 15% of CRC patients present hepatic metastasis at the time of diagnosis and 50% will develop liver metastasis after their primary resection^2^. Moreover, 30–40% of patients with advanced colorectal cancer will develop metastases to the liver only^3^, pointing out metastasis as a promising target for antitumor treatments to improve the survival of colorectal cancer patients.

Growing evidence supports a significant role for stromal cells of the host organ in the initiation and progression of the metastatic cascade^4^, and the implication of adhesion molecules in the crosstalk between tumor and host cells is currently under the spotlight^5^. Indeed, changes in the expression of adhesion molecules in the tumor cell surface facilitate tumor dissemination, colonization^6^ and metastatic growth in the liver^7^. However, the involvement of adhesion molecules from the host organ in the metastatic cascade needs further attention.

Formation of clinically relevant metastases depends on the generation of a favorable local microenvironment different from that of the neighboring tissues, which allows metastatic cell retention, survival, and growth^8^. An inflammatory response boosts tumor growth and metastasis and thus represents one of the hallmarks of cancer progression^9^. Tumor cells must interact with LSECs during early stages of liver colonization, in such a way that, as a result, a welcoming microenvironment for invading cells is generated. While the involvement of other liver-specific surface adhesion molecules such as E-selectin in liver metastasis has been reported^10^, the implications of host ICAM-1 in cancer development has remained almost unexplored. Intercellular adhesion molecule-1 (ICAM-1) is a transmembrane glycoprotein of the immunoglobulin (Ig)-like superfamily^11^ expressed constitutively in endothelial cells such as LSECs [Arteta *et al*., unpublished data]. Several inflammatory conditions enhance the expression of this molecule^12^. ICAM-1 interaction with its ligand, lymphocyte function associated 1 (LFA-1) mediates the adhesion and infiltration of leukocytes and immune cells into the hepatic tissue^13^. Furthermore, co-culture conditions induce ICAM-1 and LFA-1 upregulation in HUVECs and melanoma tumor cells, respectively^14^.

We have previously shown that activated LSECs release inflammatory factors after ICAM-1 ligation with tumor LFA-1, that correlates with a decreased lymphocyte cytotoxic activity^15^. Moreover, decreased expression of LFA-1 on tumor cells is associated with a reduction in liver metastasis of colorectal cancer in mice^16^. Indeed, a role for endothelial ICAM-1 on tumor transmigration could be envisioned as ICAM-1 ligation by LFA-1 receptor on bladder carcinoma cells leads to HUVEC cell retraction and transmigration^17^. Also, the stimulation of tumor cells with soluble ICAM-1 (sICAM-1) promotes tumor cell secretion of proangiogenic vascular endothelial growth factor (VEGF) in spheroid culture^18^. Besides, sICAM-1 stimulates tumor cell overexpression of prometastatic genes^19^. Additionally, our group has found that LSECs further contribute to the formation of a prometastatic microenvironment through their crosstalk with hepatic stellate cells (HSCs) at specific sites of circulating cancer cell arrest and transmigration across the endothelial barrier [Olaso and cols, unpublished]. An inflammatory environment promotes HSC transdifferentiation to myofibroblasts^20^. HSC-derived myofibroblasts contribute to the formation of a desmoplastic, proangiogenic stroma from the earliest state of micrometastasis formation through their secretion of migratory factors such as VEGF and metalloproteinases^21^. As a result, the interplay between tumor cells and activated LSECs and HSCs promote the transition from an early avascular micrometastatic growth to large, vascularized metastatic foci. Despite this, more studies are required to understand the mechanisms by which LSEC ICAM-1 influences the initial steps of liver metastasis while supporting and favoring disease progression. In the present study we aimed to elucidate the role of ICAM-1, produced by LSECs, on the crosstalk between tumor and host cells in early steps of liver metastasis.

## Materials and Methods

### Animals

Balb/c male mice (6–8 weeks old) were obtained from Charles River (Barcelona, Spain). Housing, care, and experimental conditions were carried out in conformity with institutional guidelines and national and international laws for experimental animal care. The animals were fed ad libitum with standard chow and water. All the proceedings were approved by the Basque Country University Ethical Committee (CEID) and by institutional, national and international guidelines regarding the protection and care of animals used for scientific purposes.

### Cancer cell lines

All *in vitro* and *in vivo* experiments were conducted using the murine C26 colon adenocarcinoma (C26) cell line (also known as MCA-26, CT-26) syngenic with Balb/c mice and purchased from ATCC (LGC Standards S.L.U. Barcelona, Spain). C26 cells were cultured under standard conditions in RPMI-1640 supplemented with 10% heat-inactivated fetal bovine serum (FBS), penicillin (100 U/ml), streptomycin (100 µl) and amphotericin B (25 µg/ml). The replacement of cells was done no later than ten passages to prevent any change in their properties.

### *In vivo* ICAM-1 silencing procedure

We used small-interfering RNAs against ICAM-1 (Life Technologies Inc; MD, USA) for the reduction of ICAM-1 expression in mice. ICAM-1 siRNA (200 ng) or scramble siRNA were diluted in sterile PBS (500 µl). The siRNA was injected in a final volume of 500 µl through the tail vein at very slow flow rate to avoid spilling. The siRNA was injected 48 and 24 hours before tumor cell inoculation. We also checked the levels of endothelial ICAM-mRNA and protein expression at the time of tumor injection. The intraperitoneal doses were given to reinforce the ICAM-1 silencing procedure. To avoid the stress generated by the procedure in awaken animals, we anesthetize the mice prior to the injection of the siRNA through the tail vein.

### Isolation and culture of primary LSECs and HSCs

The isolation and culture of mouse LSECs and HSCs have been described elsewhere^22^. Briefly, the liver was perfused with collagenase buffer from Clostridium histolyticum (Sigma-Aldrich, St. Louis, MO, USA) and the obtained cell suspension was subjected to isopycnic centrifugation through a Percoll gradient (GE Healthcare, Chicago, IL, USA). The fraction enriched in LSECs was cultured onto 1 mg/ml collagen type I (Sigma-Aldrich, St. Louis, MO, USA) coated tissue culture plates at 3′5 × 10^5^ cell/cm^2^ in RPMI-1640 media supplemented with 5% FBS, antibiotics, and antimycotics. HSCs were plated on uncoated plastic dishes. LSECs and HSCs were incubated at 37 °C, 5% CO_2_ for at least 2 hours in low serum media before any experimental use.

### Establishment of LSEC cocultures with tumor cells

Tumor cells were added on top of primary LSEC monolayers at a ratio of 1:6 and cultured with RPMI-1640 supplemented with 5% serum and antibiotics for 3 hours. Next, fresh medium supplemented with 1% serum was added, and the cells were allowed to interact for 18 hours. Then, the culture supernatant was collected. In some experiments, ICAM-1 was blocked in primary LSEC using an anti-ICAM-1 antibody for 1 hour before the addition of tumor cells. Tumor cell suspensions were incubated for 1 hour with 1 µg/ml anti-CD11a or control irrelevant antibodies (Thermo Scientific; MD, USA) before seeding them on top of LSEC monolayers.

### *In vitro* migration of primary LSEC and HSC

LSEC and HSC migration assay were carried out using Modified Boyden chambers. 2 × 10^5^ primary LSECs and HSC were seeded onto 8 μm-diameter pore membranes (Greiner Bio-one) (coated with type I collagen for LSEC culturing) and allowed to adhere and spread for 3 hours before treatment. We then treated the cells with C26 cell-derived medium or sICAM-1 activated C26 cell-derived medium for 18 hours, and the migrated cell numbers were quantified. To analyze the effect of the tumor-activated HSC-derived medium, LSECs were treated for 18 hours at different conditions. For quantification, cells were fixed in 4% formalin, stained with Dapi (Sigma-Aldrich, St. Louis, MO, USA) and counted in the microscope under 20 high-power ten fields per membrane. Data are expressed relative to the migration of control LSEC and HSC through membranes.

### Cancer cell adhesion to LSEC monolayers

C26 cells were labeled with 25 μM CFSE probe, (Thermo Scientific; MD, USA) by a 30 min incubation at 37 °C, followed by washing in the basal culture medium. Labeled cells were then resuspended to the experimental cell concentration of 2 × 10^5^ cells/ml. In some experiments, primary LSECs were incubated for 1 hour with the anti-ICAM-1 antibody (Thermo Fisher Scientific; MD, USA). In another set of experiments, LSECs freshly isolated from livers treated with ICAM-1 siRNA silencing or with an scramble siRNA were plated in basal media. Then, tumor cells were seeded onto the LSEC cultures. The resulting co-cultures were maintained for 30 min at 37 °C. Then, total emitted fluorescence was measured using Ascent Fluoroskan (Labsystems S.A.C.). Then, co-cultures were extensively but delicately washed with culture medium to prevent removing of adherent cells. The fluorescence emitted by adhered cells was again measured. Finally, the percentage of adhered cells was calculated by the subtraction of background fluorescence as follows:

$$ \% \,{\rm{adhesion}}=({\rm{fluorescence}}\,{\rm{emitted}}\,{\rm{by}}\,{\rm{adhered}}\,{\rm{C}}26\,{\rm{cells}}\times 100)/{\rm{total}}\,{\rm{fluorescence}}$$

### Transendothelial tumor cell migration assay

The transmigration, an assay that implicates that tumor cells are allowed to migrate through LSEC monolayers, was carried out using modified Boyden chambers (Greiner Bio-one). LSECs were isolated from either untreated mice or mice treated with either scramble siRNA or with ICAM-1 siRNA and cultured onto type I collagen-coated membranes of eight μm-diameter pore. LSECs were allowed to adhere for ninety minutes. Then, LSEC cultures were treated with or without ICAM-1 antibody for 1 hour, and 1 × 10^4^ tumor cells seeded on top of the LSEC monolayers. Migrated C26 cells were fixed in 4% formalin, stained with crystal violet (Sigma-Aldrich, St. Louis, MO, USA) and ten fields per membrane were counted in the microscope under 20 high-power. Data are expressed relative to the migration of C26 cells through LSECs layers from control, or scramble siRNA treated mice.

### ELISA assays

Tumor cells were seeded at a concentration of 1 × 10^5^ on top of collagen type I coated plates. After 3 hours, cells were activated with sICAM-1 (200 ng/ml) for 10 hours and with a fresh basal medium for the following 18 hours. The tumor-derived media was then collected, and VEGF, IL-1β, IL-6, TNF-α, and PGE_2_ were quantified by ELISA (Thermo Fisher Scientific; MD, USA). HSC cultures were treated with tumor-derived conditioned media for 10 hours and with a fresh basal medium for the following 24 hours. Finally, VEGF in the media was measured by ELISA.

### Gelatin and plasminogen/fibrinogen zymography

The presence of MMP-2 and MMP-9 media was determined by gelatin zymography as described previously^23^. In brief, culture supernatants were obtained from liver cells cultured under basal conditions or with media derived from C26 cell cultures, pretreated or not with sICAM-1. Cell culture supernatants were collected and run in 1% gelatin containing 10% bis-acrylamide gel. Gels were incubated overnight in developing buffer and stained with Coomassie Blue (BioRad, CA, USA). For uPA quantification, zymographies were carried out in gels containing gelatin and plasminogen. Images were taken through Quantity One program (BioRad, CA, USA).

### Cancer cell early retention and experimental development of hepatic metastasis

Tumor cells were suspended at a concentration of 2 × 10^5^ in PBS and were intrasplenically injected into anesthetized mice as previously described^15^. For retention studies, tumor cells were labeled with CFSE before the intrasplenic inoculation. After 24 hours, mice were euthanized, and the liver was removed and embedded in OCT (Tissue-Tek®, The Netherlands) before frozen in dry-ice. For hepatic metastasis, mice were sacrificed 14 days after tumor cell injection, the liver was collected and fixed in zinc-fixative solution, and paraffin embedded for histological analyses after H&E staining or embedded in OCT, frozen in dry ice, and kept at −80 °C until being processed for immunohistochemical analysis. The area of the organ occupied by the tumor cells was quantified in 10 μm thick sections. Three different regions of each liver were evaluated allowing 500 μm separation between each of them. Liver metastasis area was calculated as the area of metastatic foci per 100 mm^2^ of liver section. *In vivo* assay was performed twice with at least five mice per group.

### Immunohistochemical analysis

For the recruitment of HSCs and LSECs, the quantification of the area occupied by αSMA and CD31 expressing cell within the tumors was carried out. After blocking the samples with 5% serum containing PBS and H_2_O_2_ for 10 minutes, liver samples were incubated with the primary mouse anti-human antibody against αSMA or rat anti-mouse CD31 for 2 hours (Thermo Fisher Scientific; MD, USA). Next, samples were washed, and the secondary antibody was added to the samples. Finally, HRP was utilized for the visualization of the expression of both markers.

### Statistical analyses

Data are expressed as the mean ± standard deviation (SD) of three independent experiments. Statistical analysis was performed using SPSS version 13.0 (Professional statistic, Chicago, IL, USA). Individual comparisons were performed using two-tailed, unpaired Student t-test.

### Ethical approval

All applicable international, national, and/or institutional guidelines for the care and use of animals were followed. All the proceedings were approved by the Basque Country University Ethical Committee (CEID) and by institutional, national and international guidelines regarding the protection and care of animals used for scientific purposes.

## Results

### Involvement of endothelial ICAM-1 in tumor cell adhesion and transmigration

**Figure 1:**
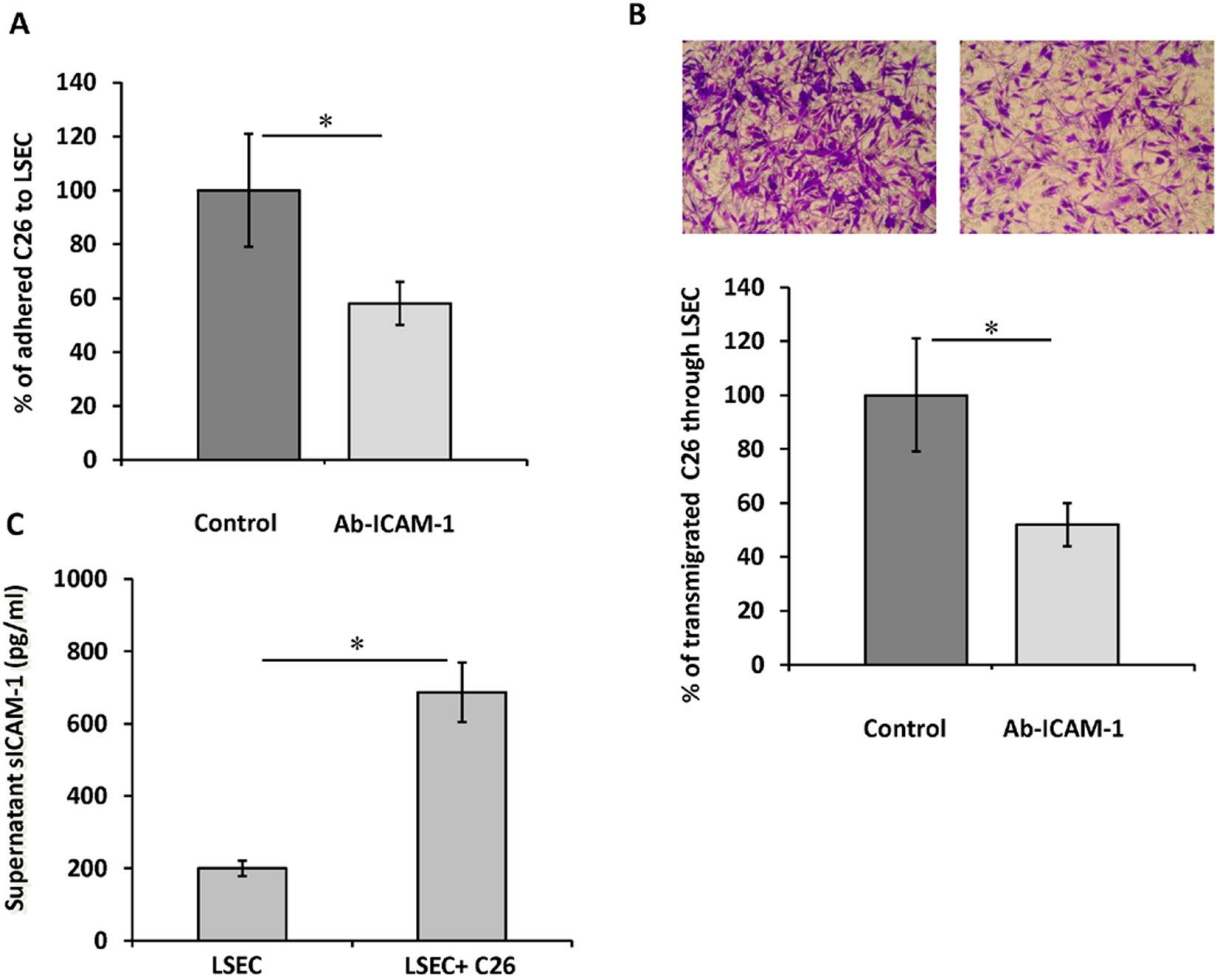
Tumor cell adhesion and transmigration through LSECs pretreated with anti-ICAM-1. (**A**) Freshly isolated mouse LSECs were treated with anti-ICAM-1 antibody or not (control) for 1 hour. Then, CFSE labeled tumor cells were added and allow to adhere for 30 minutes before total emitted fluorescence was measured. Followed by an intensive wash to remove non adherent cells, fluorescence emitted by adhered cells was measured. (**B**) Freshly isolated mouse LSECs were plated in 8 μm pore-membrane inserts. Then, tumor cells were seeded on top and allow to transmigrate through LSECs monolayer. Tumor cells transmigration across control and anti-ICAM-1 treated LSECs was quantified. Ten fields per membrane were counted in the microscope under 20 high-power for transmigrated C26 cell quantification. (**C**) Soluble ICAM-1 secretion was quantified by ELISA on the supernatants of LSECs-C26 cells cocultures. *p < 0,05.

Circulating tumor cells must adhere to the vasculature of the target organ as a first step to metastasize. In the liver, LSECs represent the major interface between the portal blood and the parenchyma. Hence, adhesion and subsequent migration of blood borne tumor cells across LSECs is a crucial event to form liver metastasis. Ligation of ICAM-1 of LSECs to LFA-1 of inflammatory cells is key to allow extravasation of the latter cells across the sinusoidal lining^13^, but its role in the tumor transmigration to the liver tissue remains unknown. To study this, we observed the extent of C26 cell adhesion to primary monolayer cultures of LSECs pretreated or not with anti-ICAM-1 antibodies. A 40% reduction in C26 cell adherence to the LSECs caused by this treatment suggested that C26 cell-LSEC ICAM-1 adhesion is mediated by ICAM-1 on LSECs (Fig. 1A).

Once tumor cells have adhered to LSECs, they must migrate across the endothelial layer to invade the liver parenchyma. Thus, next, we studied the implication of endothelial ICAM-1 in the ability of tumor cells to transmigrate across monolayers of primary cultured LSECs. Blockage of endothelial ICAM-1 reduced tumor transmigration by 50% (Fig. 1B). Furthermore, C26 cell attachment to LSECs stimulates the production of sICAM-1 by endothelial cells, amplifying ICAM-1 mediated response (Fig. 1C). In these experiments, we could not observe any detectable sICAM-1 on C26 cell supernatants (results not shown). This data, taken together, uncover a relevant role of host ICAM-1 in liver invasion by circulating tumor cells.

### Involvement of host endothelial ICAM-1 in the inflammatory cytokine profile of tumor cell/LSEC co-cultures

**Figure 2:**
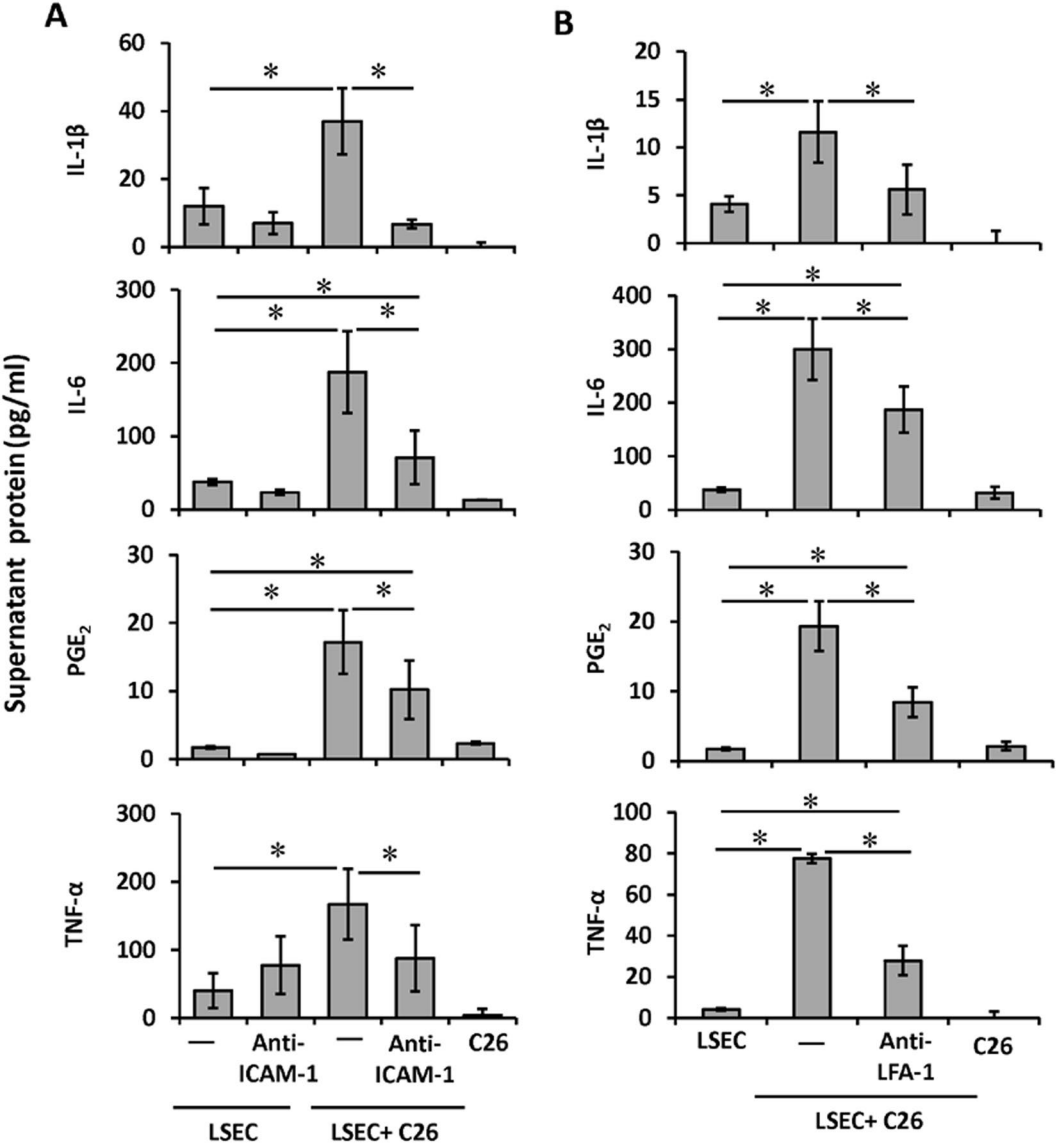
Secretion of inflammatory cytokines by tumor cell/LSEC cocultures following ICAM-1 or LFA-1 blockage in LSECs. Inflammatory cytokines were quatified in the supernatants of LSECs cocultured with C26 cells. (**A**) C26 cells were added to monolayers of LSECs either pre-treated or not with anti-CAM-1 antibody for 1 hour. (**B**) C26 cells were treated or not with antibody against LFA-1 before added to freshly solated LSECs. Supernatants from the resulting LSEC monocultures, LSEC/C26 cell cocultures and C26 cell monocultures were collected after 24 hours and the levels of IL-1β, PGE2, TNF-α and IL-6 were measured by ELISA. *p < 0,05.

Using an ELISA approach, we next examined the inflammatory cytokine profile of tumor cell/LSEC co-cultures. Media from C26 cell/LSEC co-cultures contained, on average, three-fold higher amounts of inflammatory IL-1β, IL-6, PGE_2_ and TNFα than media from LSEC monocultures (Fig. 2). To further analyze the implication of ICAM-1 in the inflammatory response generated by tumor cell interaction with LSEC during early stages of liver metastasis, we blocked ICAM-1 on LSEC primary cultures, co-cultured them with tumor cells and then determined the levels of soluble inflammatory factors in the resulting supernatants (Fig. 2A). Because LFA-1 is the principal canonical ligand for ICAM-1, in the second set of experimental conditions we blocked CD11a subunit of LFA-1 in the tumor cells with specific antibodies before co-culturing them with LSECs (Fig. 2B).

Under both experimental conditions, disrupting endothelial ICAM-1/tumor LFA-1 interaction results in a dramatic reduction in the secretion of IL-1β, IL-6, PGE_2_ and TNFα into the co-culture supernatant. These findings identify tumor LFA-1 as the primary ligand for LSEC ICAM-1, and LSEC ICAM-1/tumor LFA-1 as a critical modulator of the inflammatory switch during circulating tumor cell liver colonization.

### Tumor inflammatory and proangiogenic responses upon ICAM-1 activation

**Figure 3:**
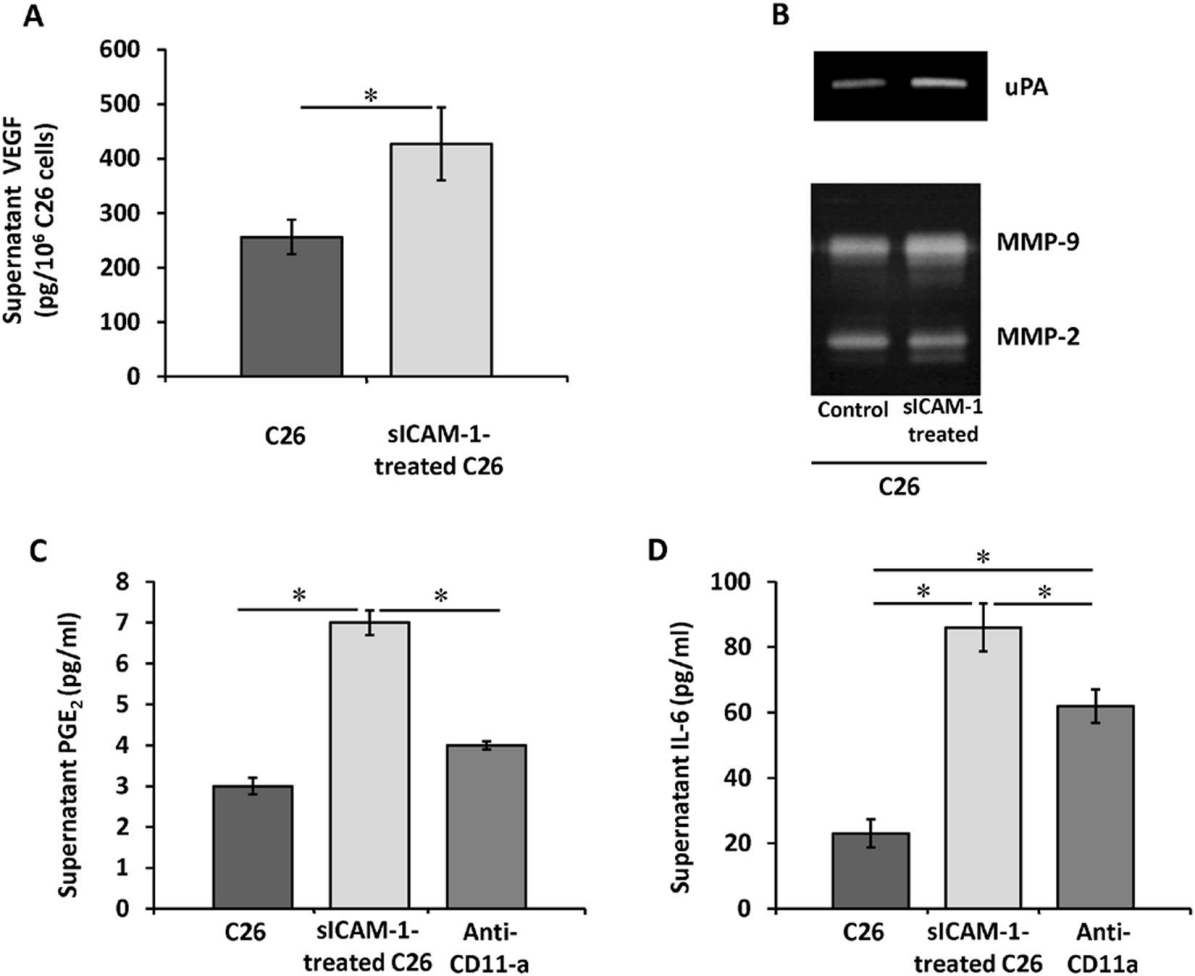
Secretion of angiogenic and inflammatory factors by ICAM-1-stimulated tumor cells. C26 cell cultures were treated or not (control) with soluble ICAM-1 for 10 hours. Afterwards, culture media were replaced by fresh media and cells were cultured for another 24 hours. The levels of VEGF, PGE2, and IL-6 were quantified by ELISA (**A,C,D**). Also, the expression of uPA, MMP-2 and MMP-9 by zymography were analyzed in sICAM-1- stimulated C26 cells (**B**). *p < 0,05.

Given that LSECs secrete sICAM-1 as a response to C26 cell adherence (Fig. 1C), in the next set of experiments we aimed to determine whether sICAM-1 modifies tumor cell release of pro-metastatic soluble factors enabling tumor foci formation and growth. Exposure of C26 cells to sICAM-1 increased the production of VEGF, PGE_2_ and IL-6 in C26 cell monocultures by factors of 1.8, 2.7 and 4, respectively (Fig. 3A,C,D), compared to untreated monocultures. Moreover, sICAM-1 exposure also promoted tumor secretion of matrix remodeling factors uPA, MMP-2, and MMP-9 (Fig. 3B). As a whole, these results indicate that the activation of tumor cells by sICAM-1 enhance tumor secretion of proliferative, proangiogenic and matrix remodeling factors implicated in the metastatic development in the liver.

### Soluble factors from ICAM-1- activated tumor cells modulate the migration of primary LSECs and HSCs

**Figure 4:**
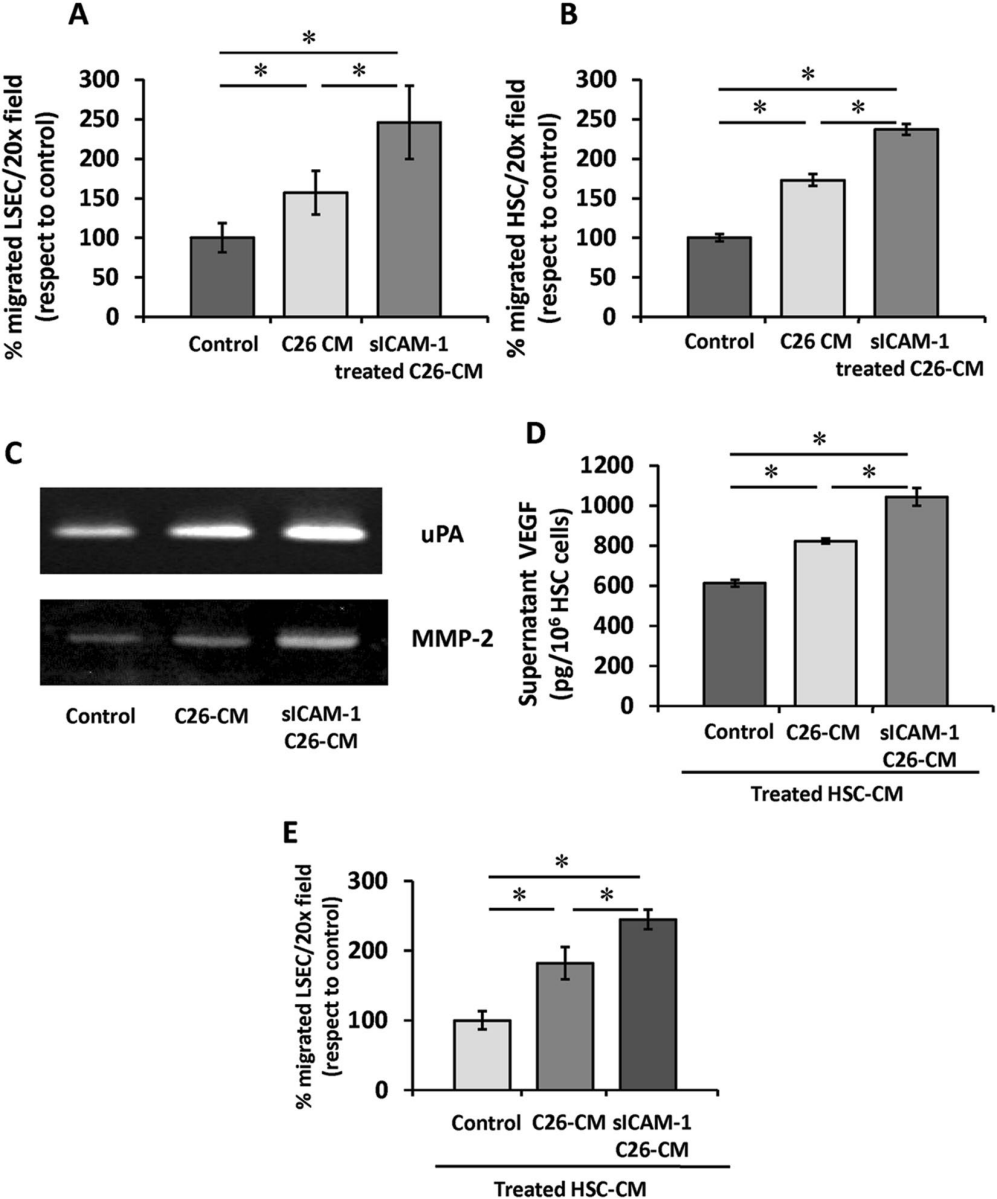
Migration of primary LSECs and HSCs in response to soluble factors secreted by ICAM-1- activated tumor cells (**A**,**B**). Freshly isolated mouse LSECs (**A**) and HSCs (**B**) were cultured on top of 8 μm pore membrane inserts and allowed to migrate for 24 and 36 hours, respectively towards the supernatants derived from untreated or sICAM-1 stimulated C26 cells. Migrated cells stained with DAPI were counted in 10 20x-fields per well. *p < 0,05. Secretion of angiogenic factors by HSCs activated by sICAM-1-stimulated tumor cells (**C**–**E**). Tumor cell cultures were treated or not (control) with soluble ICAM-1 for 10 hours. Primary HSCs cultures were activated with supernatants from control or ICAM-stimulated tumor cell cultures. The media was changed for fresh medium, collected after 24 hours and analyzed for uPA, MMP-2 by zymography (**C**) and by ELISA for VEGF content (**D**). (**E**) Primary LSECs were seeded in 8 μm poremembranes and treated with tumor-activated HSC culture supernatants. Migrated cells stained with DAPI were counted in 10 fields per well. *p < 0,05.

Tumor growth requires the recruitment of supporting stromal cells. Thus, we studied the migratory potential of freshly isolated LSECs and HSCs in response to soluble factors secreted by sICAM-1 activated tumor cells. As illustrated in Fig. 4A, tumor-derived soluble factors promoted migration of LSECs 1.5-fold more than basal media. Migration of LSECs in response to supernatants from sICAM-1 pre-stimulated tumor cells was 2.5 fold higher than with basal media. Consistent with previous results^23^, HSCs cultured in the presence of tumor supernatants migrate 1.7-fold more than under basal conditions. Migration of HSCs after exposure to supernatants from sICAM-1 pre-stimulated tumor cells was 2.4 times higher than basal media (Fig. 4B). Therefore, tumor activation by sICAM-1 enhances the recruitment of LSECs and HSCs to the proximity of tumor cells invading the sinusoids.

### Effect of ICAM-1 activation of tumor cells on the angiogenic potential of HSCs

Tumor cells promote HSC activation to a proangiogenic and desmoplasic myofibroblastic phenotype characterized by increased or *de novo* synthesis and secretion of proangiogenic VEGF and ECM remodeling proteases^21^. To analyze the significance of ICAM-1 in tumor-mediated HSC activation, freshly isolated HSCs were treated with tumor supernatants. Zymography analysis showed that HSCs cultures contained a higher expression of uPA and MMP-2 when exposed to tumor supernatants, and supernatants from C26 cells activated with sICAM-1 further promoted HSCs expression of these proteases (Fig. 4C). Similarly, VEGF secretion was elevated by 33% in HSCs cultured in the presence of tumor supernatants and further increased to 60% in HSCs treated with supernatants from sICAM-1 pre-treated tumor monocultures (Fig. 4D).

Tumor-activated HSCs facilitate LSECs migration *in vitro*^21^. In concordance with this, the supernatants from tumor activated HSCs increased the number of migrated LSECs by almost 1.7-fold compared to supernatants from untreated HSCs (Fig. 4E). This promigratory effect was enhanced up to 2.4-fold by supernatants from HSCs previously activated by supernatants collected from sICAM-1 pre-treated C26 cell cultures (Fig. 4E). In summary, supernatants from ICAM-1 activated C26 cells promote a proangiogenic phenotype in HSCs.

### Effect of sICAM-1 or anti-CD11a antibody pretreatment of tumor cells on their pro-metastatic properties

**Figure 5:**
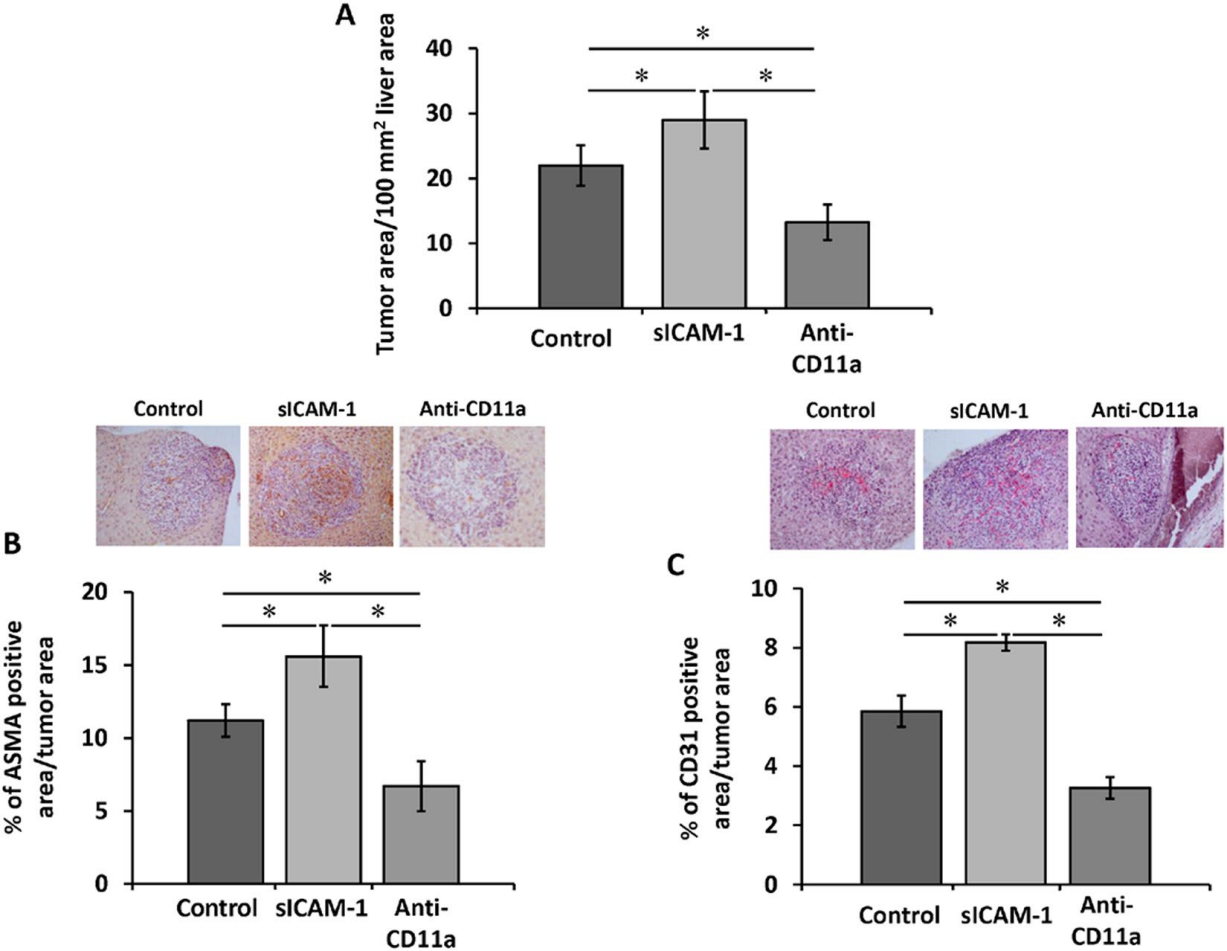
Effect of tumor cell treatment with sICAM-1 or with anti-CD11a antibodies in liver metastasis and angiogenesis. Tumor cells were treated *in vitro* with sICAM-1 or with anti-CD11a antibodies or led untreated before injection. Mice were intrasplenically injected with tumor cells. Fourteen days afterwards livers were analyzed for metastatic area (**A**), αSMA expression (**B**) and CD31 (**C**) expression. Three liver sections per mice, each one separated by 500 μm from the other were analyzed *p < 0,05.

Our *in vitro* data suggests that ICAM-1 modulates tumor cell migration and release of pro-migratory factors to recruit LSECs and HSCs. Thus, we next utilized our *in vivo* model of liver metastasis to analyze how *in vitro* alteration of tumor cell response to ICAM-1 modifies tumor ability to develop liver metastasis. Tumor cells were subjected *in vitro* to either stimulation with sICAM-1 or blockage of LFA-1 binding capacity to ICAM-1 with anti-CD11a antibody, prior to injecting them intrasplenically to syngenic mice. Fourteen days later, we observed a significant difference in the metastatic burden in the livers between the two experimental groups. *In vitro* sICAM-1 pre-stimulation of tumor cell resulted in a 35% higher metastatic colonization area of the liver than what we observed in livers receiving untreated tumor cells (Fig. 5A). On the contrary, the ability of anti-CD11a treated tumor cells to metastasize to the liver was found to be reduced by 50% in metastatic area in the liver compared to livers receiving untreated tumor cells, and by 65% when compared to metastasis generated by sICAM-1 pretreated tumor cells (Fig. 5A). These differences in the metastatic growth rate between the two experimental groups correlated with altered intratumoral contents of αSMA- and CD31-expressing cells. Intratumor liver foci neovessels contained activated myofibroblastic HSCs, detected by their *de novo* αSMA expression, and CD31-expressing activated LSECs^21^. Both αSMA and CD31 expression increased by 30% and 25%, respectively, in livers collected from mice bearing sICAM-1 treated tumor cells, indicating a higher number of activated HSCs and angiogenic LSECs in tumor foci originated from sICAM-1 treated tumor cells (Fig. 5B,C). As expected from our previous *in vitro* data, the livers collected from mice injected with tumor cells pretreated with anti-CD11a showed both reduced area occupied by metastatic foci and a 40% decrease in foci infiltration by activated HSCs and LSECs (Fig. 5B,C). Altogether, these results indicate that endothelial ICAM-1/tumor LFA-1 interplay facilitates liver metastasis through the recruitment of a prometastatic stroma composed of activated HSCs and angiogenic LSECs.

### Effect of *in vivo* ICAM-1 silencing on tumor retention in the liver. *In vitro* tumor cell adhesion and transmigration through LSEC monolayers from ICAM-1 silenced mice

**Figure 6:**
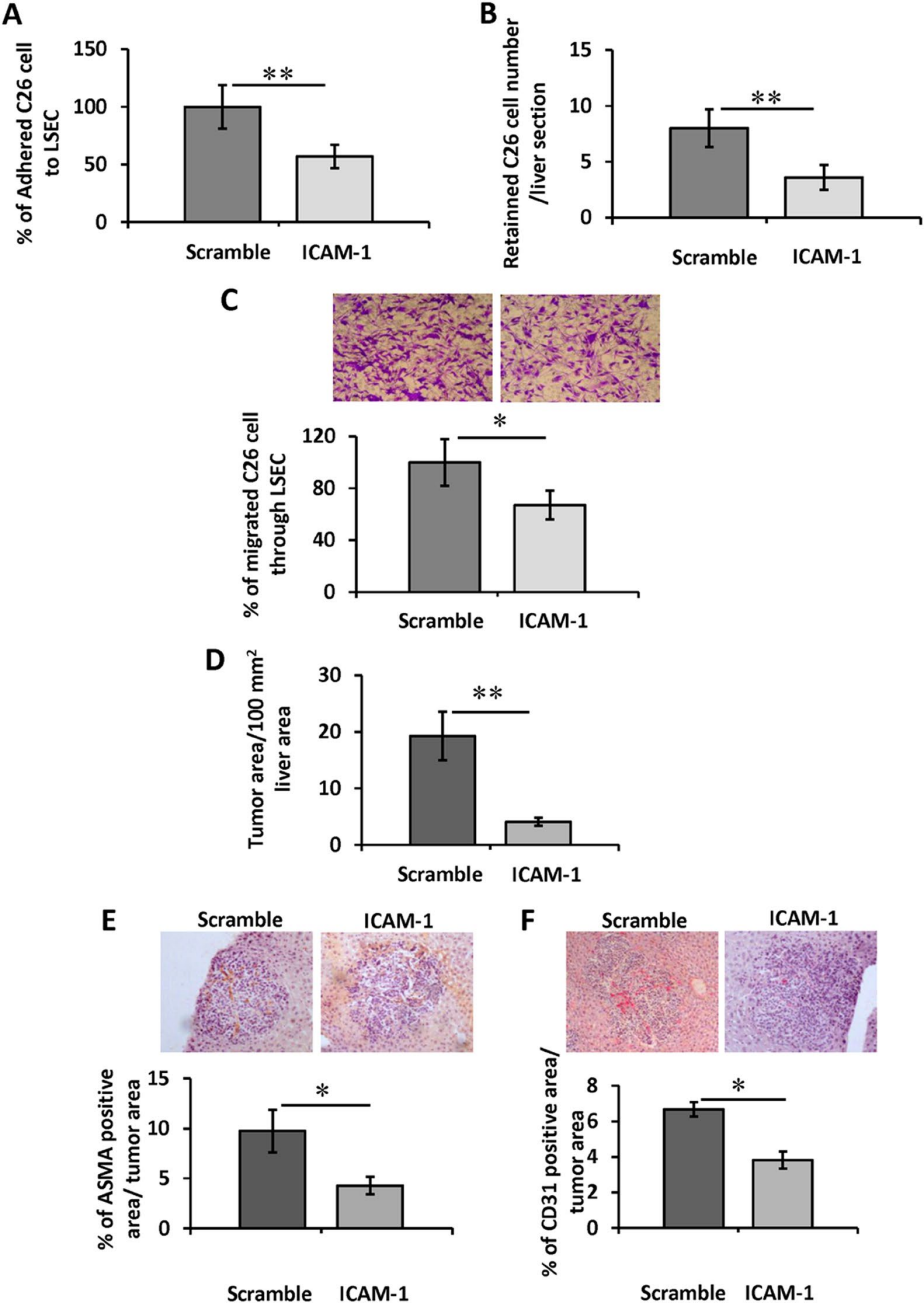
Effect of *in vivo* silencing of ICAM-1 in the *ex vivo* tumor cell adhesion and transmigration through LSEC monolayers and *in vivo* tumor cell retention in the liver, liver metastasis and angiogenesis. Mice were injected with scramble siRNA or ICAM-1 siRNA through the tail vein. (**A**) 24 hours afterwards, LSEC were isolated and cultured for 3 hours. Then, CFSE-labeled tumor cells were added and allowed to adhered for 20 min. Then, tumor cell adhesion was quantified as shown before. (**B**) To quantify *in vivo* tumor cell retention to the liver, CFSE-labeled were i.s. injected into scramble and ICAM-1 siRNA treated mice. Livers were collected 24 hours after tumor cell injection and embedded in OCT. Retained tumor cells were quantified in 3 different 10 μm–thick liver sections/liver separated by 500 μm (n = 4 mice per group). (**C**) To quantify tumor cells-transmigration through LSECs, they were shed on top of LSEC monolayers cultured on top of 8 μm pore membrane inserts. Transmigrated tumor cells were stained with DAPI and count in 10 20x -fields per well. *p < 0,05, **p < 0,01. For metastasis development assay, mice were injected with scramble siRNA or ICAM-1 siRNA through the tail vein. Then, mice were injected intrasplenically with tumor cells. Fourteen days afterwards livers were analyzed for metastatic area (**D**), αSMA expression (**E**) and CD31 (**F**) expression. Three liver sections per mice, each one separated by 500 μm from the other were analyzed *p < 0,05.

Our *in vitro* and *in vivo* data point out ICAM-1 as a mediator of the inflammatory and angiogenic host responses during the early stages of liver metastasis. To further confirm these findings *in vivo*, we silenced LSEC ICAM-1 by siRNAs injection and then checked for *in vitro* adhesion and transmigration of tumor cells to LSECs isolated from those mice (See silencing efficiency in Suppl. Fig. 1). In concordance with our previous results using ICAM-1 antibodies (Fig. 1), adhesion of tumor cells to ICAM-1 silenced LSECs was 2-fold lower than tumor adhesion to LSECs expressing ICAM-1 derived from mice injected with scramble siRNA (Fig. 6A). The involvement of ICAM-1 in tumor cell adhesion to the hepatic sinusoidal wall was confirmed by *in vivo* retention assays. One day after intrasplenic inoculation, tumor cells are already retained in the liver^16^. At this time point, ICAM-1-silenced livers contain 50% fewer tumor cells than livers from mice injected with scramble siRNA (Fig. 6B). In concordance with these results, *ex vivo* transendothelial migration of tumor cells through freshly isolated LSECs from mice injected with siICAM-1 was 35% less than transmigration across LSECs from mice injected scramble siRNA (Fig. 6C).

### Effect of *in vivo* ICAM-1 siRNA injection to mice in liver metastasis and angiogenesis

To further analyze the *in vivo* effect of reduced ICAM-1 expression in the liver in tumor formation, in the next set of experiments we examined metastatic tumor foci formation and growth in mice with silenced expression of LSEC ICAM-1. To do so, we injected mice with ICAM-1 siRNA 48 and 24 hours before tumor cell injection and three and six days after. Fourteen days after tumor cell-injection, histological analyses of the livers showed a drastic 75% drop in the area of liver tissue occupied by metastatic foci compared to livers from scramble siRNA treated mice (Fig. 6D). Tumor foci of ICAM-1 siRNA treated mice contained 55% less αSMA and 45% CD31 expression than control ones (Fig. 6E,F).

## Discussion

ICAM-1 expression on tumor cells has been extensively studied and linked with their ability to invade adjacent and distant organs and with their increased metastatic potential in osteosarcoma, breast or colorectal cancer cells^24–26^. In this work, we report a crucial role for endothelial ICAM-1 in the *in vitro* C26 cell adhesion and transmigration through LSECs and the *in vivo* formation of a prometastatic inflammatory and angiogenic microenvironment. To our knowledge, this is the first report on the role of hepatic endothelial ICAM-1 on the early steps of liver colonization by CRC cells. We use several approaches to reinforce the strength of our findings. First, we use freshly isolated sinusoidal cells. As far as we know, this is the first report on ICAM-1 dependent tumor transmigration through freshly isolated LSECs and HSCs. The use of such cells is relevant because it resembles more closely what may happen *in vivo*. Second, we use several *in vivo* and *in vitro* approaches, such as *in vitro* blockage of ICAM-1/LFA-1 interaction by antibodies and *in vivo* iCAM-1 silencing, to analyze how ICAM-1 signals in LSECs. Each of them reinforced the others and allow us to conclude that host liver sinusoidal endothelial ICAM-1 is a mediator of the metastatic cascade in the liver.

Patients who suffer from colorectal cancer exhibit high serum levels of inflammatory mediators such as IL-1β, IL-6, TNF-α and PGE_2_^27,28^. In our experimental model, enhanced secretion of IL-1β, PGE_2_, TNF-α, and IL-6 occurs following CRC cells/LSECs crosstalk in a tumor LFA-1/LSEC ICAM-1 interaction dependent fashion. C26 cells stimulation by either sICAM-1 administration or co-culture with LSECs results in tumor cell secretion of inflammatory PGE_2_ and IL-6, VEGF, uPA, MMP-2, MMP-9. Co-culture conditions also enhanced LSEC secretion of IL-1β, PGE_2_, TNF-α, and IL-6. In our hands, C26 cell cultures, treated or not with sICAM-1, do not contain detectable levels of TNFα or IL-1β, while LSECs do not secrete detectable levels of VEGF, uPA, MMP-2 or MMP-9. Enhanced PGE_2_ secretion in the C26 cell/LSECs co-cultures goes along with our previous studies, where we demonstrate that ligation of tumor LFA-1 with endothelial ICAM-1 drives COX-2 mediated LSECs secretion of IL-1β^15^. Furthermore, IL-6, and TNF-α secretion is also a COX-2 dependent event in LSECs (Arteta personal communication). In our model VEGF production is partially ICAM-1 dependent, which may explain why LSEC ICAM-1/tumor LFA-1 crosstalk modulates LSEC migration *in vitro* and LSEC angiogenic response inside the tumor foci.

Invading tumor cells recruit stromal cells to promote tumor growth. In the liver, HSCs and LSECs contribute to the angiogenic switch from avascular metastasis to clinically relevant, angiogenic ones. HSCs are one of the first cell types arriving into the proliferating tumor cell niche^23^. Using a melanoma model, we have shown that the crosstalk between the tumor cell and HSCs increases the release of pro-migratory factors for LSECs such as VEGF^21^. In this work, we found that soluble factors released from both tumor cells and HSCs in response to sICAM-1 favor LSECs migration. It is tempting to speculate that VEGF released by both C26 cells and HSCs following C26 cell ICAM-1 activation accounts for the enhanced movement of LSECs. Furthermore, the local environment generated by LSEC ICAM-1/tumor LFA-1 binding may directly contribute to HSCs activation. To this regard, IL-6 promotes HSC expression of myofibroblastic features such as αSMA and collagen I deposition^28,29^. ECM remodeling accompanies tumor and host cell migration. Herein, LSEC ICAM-1 mediates ECM remodeling by C26 cells and HSC-derived myofibroblasts. Excessive degradation of the matrix is one of the hallmarks of metastasis, and uPA, MMP-2, and MMP-9 play a role in human colorectal cancer progression, invasion, and metastasis^30^. IL-1β and IL6 upregulate MMP-2 and uPA in lung and gastric cancer^31–35^. Thus, our *in vitro* observations may reveal a direct link between LSEC ICAM-1 and matrix remodeling during liver colonization of CRC.

As expected from our *in vitro* data, metastatic area and recruitment of LSECs and HSCs to the tumor foci was lower in C26 cell metastasized livers collected from ICAM-1 siRNA treated mice or from C26 cells pretreated with CD11a antibodies, in line with our groups previous studies disrupting the LSEC ICAM-1/C26 cell LFA-1 crosstalk through β2 integrin blocking^16^. On the contrary, C26 cells stimulated by sICAM-1 developed more metastasis. These results are coherent with the present ones confirming the role of the interaction between host ICAM-1 and tumor LFA-1 in the development of liver metastasis.

Altogether, our results point out host endothelial ICAM-1 as a potent inflammatory and angiogenic promoter that mediates the infiltration of tumor cells and stromal cell recruitment into the tumor mass. These hosts ICAM-1 mediated tumor/endothelial crosstalk initiates inflammatory and angiogenic responses driving to liver metastasis.

## Supplementary information

Dataset 1

## Data Availability

The datasets generated during and/or analysed during the current study are available from the corresponding author on reasonable request.
