# Supplementary material for: Liver sinusoidal endothelial cell ICAM-1 mediated tumor/endothelial crosstalk drives the development of liver metastasis by initiating inflammatory and angiogenic responses
Source: Sci Rep. 2019 Sep 11;9:13111. doi: 10.1038/s41598-019-49473-7 (PMC6739321; doi:10.1038/s41598-019-49473-7)

Liver sinusoidal endothelial cell ICAM-1 mediated tumor/endothelial crosstalk drives the development of liver metastasis by initiating inflammatory and angiogenic responses

Aitor Benedicto[1,*], Alba Herrero[1], Irene Romayor[1], Joana Marquez[1], Bard Smedsrod[2], Elvira Olaso[1] and Beatriz Arteta[1]

Supplementary Dataset 1

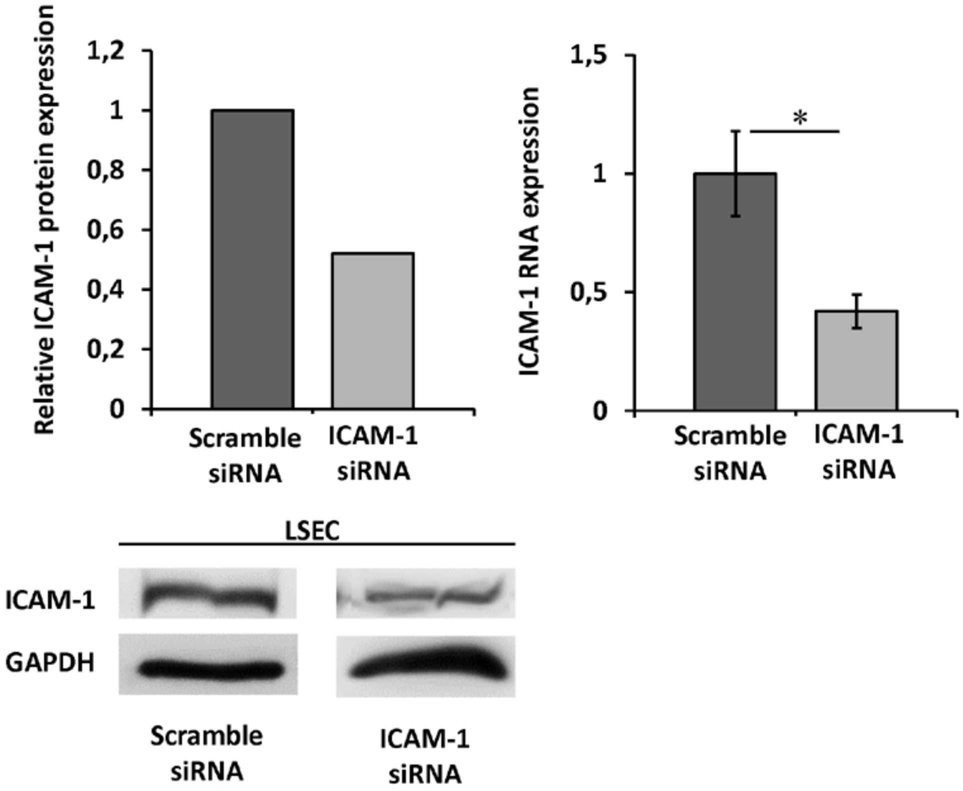

Supplement: Supplementary file 1 — Dataset 1 [file 41598_2019_49473_MOESM1_ESM.pdf]
